# Drug Discovery and Repurposing Inhibits a Major Gut Pathogen-Derived Oncogenic Toxin

**DOI:** 10.3389/fcimb.2019.00364

**Published:** 2019-10-25

**Authors:** Paul Metz, Martijn J. H. Tjan, Shaoguang Wu, Mehrosh Pervaiz, Susanne Hermans, Aishwarya Shettigar, Cynthia L. Sears, Tina Ritschel, Bas E. Dutilh, Annemarie Boleij

**Affiliations:** ^1^Centre for Molecular and Biomolecular Informatics, Radboud University Medical Center (Radboudumc), Nijmegen, Netherlands; ^2^Department of Pathology, Radboud Institute for Molecular Life Sciences, Radboud University Medical Center (Radboudumc), Nijmegen, Netherlands; ^3^Division of Infectious Diseases, Department of Medicine, Johns Hopkins University School of Medicine, Baltimore, MD, United States; ^4^Theoretical Biology and Bioinformatics, Utrecht University, Utrecht, Netherlands

**Keywords:** computational drug design and discovery, bacterial toxin, colorectal cancer, inflammatory bowel disease (IBD), *Bacteroides fragilis* enterotoxin, enterotoxigenic *Bacteroides fragilis* (ETBF), drug screening, chenodeoxycholic acid

## Abstract

**Objective:** The human intestinal microbiome plays an important role in inflammatory bowel disease (IBD) and colorectal cancer (CRC) development. One of the first discovered bacterial mediators involves *Bacteroides fragilis* toxin (BFT, also named as fragilysin), a metalloprotease encoded by enterotoxigenic *Bacteroides fragilis* (ETBF) that causes barrier disruption and inflammation of the colon, leads to tumorigenesis in susceptible mice, and is enriched in the mucosa of IBD and CRC patients. Thus, targeted inhibition of BFT may benefit ETBF carrying patients.

**Design:** By applying two complementary *in silico* drug design techniques, drug repositioning and molecular docking, we predicted potential BFT inhibitory compounds. Top candidates were tested *in vitro* on the CRC epithelial cell line HT29/c1 for their potential to inhibit key aspects of BFT activity, being epithelial morphology changes, E-cadherin cleavage (a marker for barrier function) and increased IL-8 secretion.

**Results:** The primary bile acid and existing drug chenodeoxycholic acid (CDCA), currently used for treating gallstones, cerebrotendinous xanthomatosis, and constipation, was found to significantly inhibit all evaluated cell responses to BFT exposure. The inhibition of BFT resulted from a direct interaction between CDCA and BFT, as confirmed by an increase in the melting temperature of the BFT protein in the presence of CDCA.

**Conclusion:** Together, our results show the potential of *in silico* drug discovery to combat harmful human and microbiome-derived proteins and more specifically suggests a potential for retargeting CDCA to inhibit the pro-oncogenic toxin BFT.

## Importance

Using *in silico* drug discovery and complementary *in vitro* analyses, chenodeoxycholic acid (CDCA) was found to inhibit the major gut pathogen-derived oncogenic *Bacteroides fragilis* toxin (BFT). Thus, CDCA may be a promising drug that limits ETBF-related inflammatory bowel disease (IBD) and colorectal cancer (CRC) development. CDCA is especially interesting as a BFT-inhibitor since it is one of several naturally occurring bile acids with gut concentrations well within the dose to effectively inhibit BFT. Hence, inter-individual variation in bile-acid concentrations could explain differences in the human colon response to ETBF and the risk for cancer. Moreover, CDCA has additional anti-inflammatory properties via activation of the Farnesoid X Receptor (FXR) and is an interesting compound to explore in IBD-patients. The newly discovered interaction between CDCA and BFT offers new opportunities in battling ETBF-related CRC and IBD, brings an additional option for experimental manipulation by “switching off” BFT and opens new avenues for understanding host-microbiome interactions.

## Introduction

In 2018, an estimated 861,663 people died from CRC globally, i.e., >9% of the total cancer deaths in that year (Bray et al., 2018). Over the last decade, the importance of microbial factors from our gut microbiome in CRC pathogenesis has emerged. An important CRC-driving bacterium identified in the mucosa of more than 80% of CRC-patients is enterotoxigenic *Bacteroides fragilis* (ETBF) carrying the *Bacteroides fragilis* toxin (BFT), also known as fragilysin (Boleij et al., 2015). ETBF has been specifically associated with diarrhea, inflammatory bowel disease (IBD) and anaerobic bacteremia (Wexler, 2007; Rabizadeh and Sears, 2008; Housseau and Sears, 2010; Zamani et al., 2017). ETBF is often found together with *E. coli* carrying the oncotoxin colibactin in mucosal biofilms of Familial Adenomatous Polyposis (FAP). These two oncogenic bacteria have been shown to synergize *in vivo*, creating a favorable environment for CRC development (Sears and Garrett, 2014; Dejea et al., 2018). Exposure to BFT is therefore proposed as an important microbiome-derived risk factor for CRC development in humans.

ETBF contains a 6 kilobase pair (kbp) pathogenicity island within a 40 kbp conjugative transposon that harbors the *bft* gene, encoding a 397 amino acid zinc-dependent metalloprotease (Moncrief et al., 1995; Wexler, 2007). BFT induces the degradation of the intercellular protein E-cadherin (Wu et al., 1998) of colonic epithelial cells (CECs), causing morphological changes *in vitro* such as rounding, swelling, and cluster dissolution (Rhee et al., 2009). Moreover, BFT activates the NF-B and β-catenin/Tcf signaling pathways, resulting in the expression of proinflammatory IL-8 and the c-myc oncogene, which have been associated with CRC (Wu et al., 2004; Ning et al., 2011).

BFT is the only recognized virulence factor of ETBF and considered to be fully responsible for its pathogenicity. Targeted BFT inhibition could therefore limit ETBF-related pathogenicity. Three different BFT isoforms have been identified, *bft*-1, *bft*-2, and *bft*-3 (Kato et al., 2000), whose encoded amino acid sequences are >93% identical (Chung et al., 1999; Kato et al., 2000).

BFT is encoded as a protoxin, where the active site responsible for the pro-oncogenic activity is completely blocked by a 9 amino acid pro-domain (Franco et al., 2005; Goulas et al., 2011). Below, we combine two *in silico* drug discovery techniques, drug repositioning and molecular docking, to predict potential inhibitors for BFT that interfere with its toxic activity by blocking the active site. The strongest *in silico* predicted inhibitors were tested in a CEC-model. We show for the first time that *in silico* drug prediction is particularly well suited to identify compounds effectively targeting microbial toxins.

## Materials and Methods

### *In silico* Screening to Identify BFT Inhibitors

**Figure 1:**
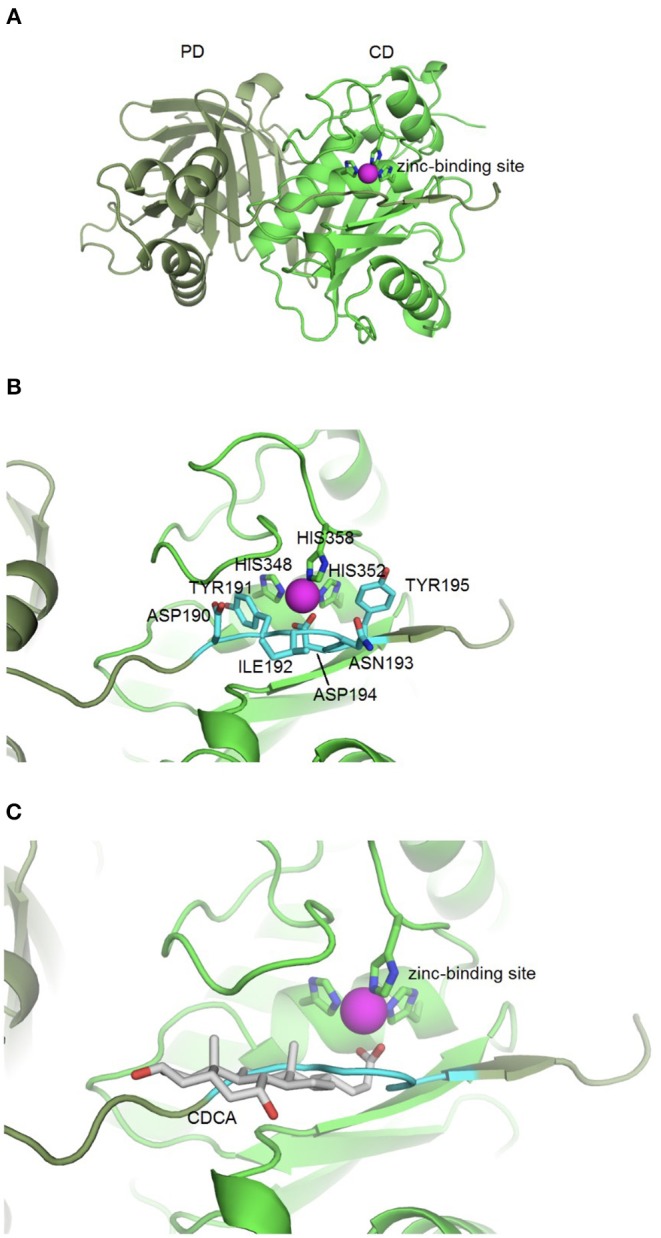
BFT-3 structure. **(A)** BFT structure and active site BFT-3 structure including pro-domain (PD, dark green) and catalytic domain (CD, light green) and zinc-binding site (zinc in purple). **(B)** Pro-domain residues (ASP190, TYR191, ILE192, ANS193, ASP194, and TYR195) used for defining the BFT-3 binding pocket (light blue) and zinc coordinating residues from the CD (HIS348, HIS352, and HIS358). **(C)** Chenodeoxycholic acid docked into the BFT-3 zinc-binding site using MOE.

Based on the idea that similar binding sites can bind similar ligands, the *in silico* structure-based pharmacophore fingerprint comparison method KRIPO (Wood et al., 2012) was used to screen the Protein Data Bank (www.rcsb.org) (Berman et al., 2000) for proteins with a structurally similar binding site as BFT-3, whose crystal structure has been resolved (PDB ID: 3P24) (Goulas et al., 2011). The catalytic site of BFT-3 is shown in Figures 1A,B: a zinc ion is complexed by three histidine residues of the catalytic domain (HIS348, HIS352, HIS358) and one aspartate (ASP194) from the pro-domain.

After cleavage of the pro-domain, the active site of the protein is exposed. To define the binding site of BFT-3, atoms ≤6 Å from the active site blocking residues (ASP190, TYR191, ILE192, ANS193, ASP194, and TYR195) of the uncleaved BFT-3 pro-domain were selected and their pharmacophore features (aromatic interactions, hydrogen bond acceptors and donors, hydrophobic contacts, and positive/negative charges) described (Figure 1B). All pharmacophore features of this binding pocket ≤2.5 Å of any atom of the previously mentioned pro-domain residues were defined as the final protein structure-based pharmacophore of the BFT-3 binding site, which was then encoded into a fingerprint bit string representation (Wood et al., 2012). With a fingerprint bit string similarity of ≥0.45 to this structure-based pharmacophore of the BFT-3 binding site, all (sub)binding pockets and their associated ligands were selected from the Protein Data Bank.

**Figure 2:**
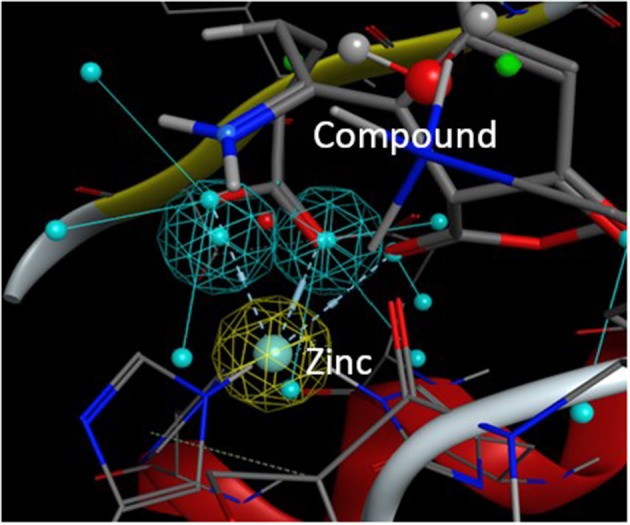
Pharmacophore model BFT-3 active site. Docking and interaction of a compound (sticks) with the BFT-3 active site (lines) guided by a pharmacophore model of the BFT-3 active site, including a pharmacophore description of the zinc atom (spheres).

From this preselection by KRIPO, ligands were extracted and subsequently docked into the BFT-3 binding site using MOE 2013.0802^1^ (Figure 1C). The docking was guided by a pharmacophore model (Figure 2) of the BFT binding site to select compounds that could interact with the zinc atom. This additional docking step, using a pharmacophore description of the zinc interactions, was essential because KRIPO does not directly encode for metal interactions.

### Cell Culture

HT29/c1 CECs were maintained in DMEM (Gibco/BRL 12800) supplemented with 10% Fetal Bovine Serum (FBS), 44 mM NaHCO_3_, 10 μg/mL Human apo-transferrin (Sigma T1147), 50 units/mL penicillin and 50 μg/mL streptomycin with pH 7.4 (Weikel et al., 1992; Sears, 2001). For morphology assays and subsequent ELISA's, HT29/c1 cells were grown in 96-well plates to a confluence of 70–80%.

### BFT Preparation

Wild type ETBF strains VPI 13784 (BFT-1), 086–5443-2–2 (BFT-2) and k570 (BFT-3) were grown anaerobically in 3.7% BHI supplemented with 0.5% yeast extract, 0.05% cysteine HCL, 10 μL/mL hemin and 0.2 μL/L vitamin K, at 37°C for 24 h. After adjusting to get approximately equal optical densities of 0.8-1.0 (*A*_600_), pooled supernatants were sterilized by filtration through a 0.2 μm filter and concentrated 5–10-fold using a 10 kDa filter (Millipore). The concentration of these concentrated toxins was calibrated using HT29c1 morphological assays (Supplementary Figure 1). Concentrated toxins were stored at −20°C until further use.

### Morphological Assay

For initial examination of the putative BFT inhibitors, BFT-induced morphological changes were assessed with a previously described *in vitro* toxin activity assay (Weikel et al., 1992). HT29/c1 cells were exposed to BFT-3 at a final calibrated concentration of 100 pM and to each of the selected compounds, at 850 nM or 8.5 μM concentrations dissolved in DMEM (total volume was 200 μL) (Table 1 and Supplementary Table 1).

**Table 1:** The twelve *in silico* selected compounds predicted to inhibit BFT.

**Abbreviation**	**Full name**
ADP-DSH	Adenosine 5′-Diphosphate Disodium Salt Hydrate
ADP-MPSD	Adenosine 5′-diphosphate monopotassium salt dihydrate
CDCA	Chenodeoxycholic acid
CoA	Coenzyme A hydrate, = 85% (UV, HPLC)
DS-DHP	Disodium 2,3-dihydroxypropyl phosphate
EBSHIPA	2-(4-Ethoxy-benzenesulfonylamino)-3-(1H-indol-3-yl)-propionic acid
FMN/SS	Riboflavin 5′-Monophosphate Sodium Salt
HEPES	2-[4-(2-Hydroxyethyl)-1-piperazinyl]ethanesulfonic Acid
PDS	Phosphoramidon disodium salt
PIPPS	Piperazine-n,n′-bis (3-propanesulfonic acid)
PPAA	[4-(1H-pyrazol-1-yl)phenyl]acetic acid
Ubenimex	Ubenimex

After this first *in vitro* screening, promising compounds were tested further at higher concentrations (85 and 175 μM). Compounds EBSHIPA, PPAA, CDCA, and DS-DHP were dissolved in 100% dimethyl sulfoxide (DMSO) and all other compounds in deionized water (dH_2_O). Positive controls contained BFT-3 (100 pM) without compounds, but with DMSO/H_2_O. Negative controls included either compound only, DMSO/H_2_O or neither. HT29/c1 cells were pre-incubated with the selected compounds in DMEM at 37°C for 30 min, before addition of BFT-3. Morphology changes were scored on a scale from 0 to 4 (Supplementary Figure 2), by 2 individual observers, every hour for a maximum of 4 h. Scores of all testing conditions were compared to positive and negative controls by using the independent unpaired *t*-test. Compounds that demonstrated BFT inhibition, were tested further in morphology assays with BFT-1 (200 pM) and BFT-2 (300 pM). The supernatants collected in these experiments were used to measure E-cadherin and IL-8 release by ELISA after 3 and 4 h respectively (Supplementary Method 1).

### Thermal Shift Assay

Protein Thermal Shift dye (Thermofisher) 2.5 μL was mixed with 5 μL of Protein Thermal Shift buffer, 1 μg of recombinant BFT-1 (Supplementary Method 1, Supplementary Table 2) in Buffer B (20 mM Tris-HCl, 150 mM NaCl and pH 7.4) and different concentrations of CDCA (0, 8.5, 85, 175, and 850 μM) (Goulas et al., 2011). Negative controls contained only CDCA [ligand only control (LOC)] or only buffer and dye [No-protein control (NPC)]. Reagents were added to a MicroAmp Optical Reaction plate spun at 1000 rpm for 1 min, and incubated in the 7500 Fast SDS Real-Time PCR system (Applied Biosystems), starting at 25°C, at a ramp rate of 1% until 99°C. Melting curves were generated by capturing fluorescent signal ROX after every cycle of temperature increase. First the raw fluorescence data were plotted in GraphPad Prism 6.00 and the post-peak fluorescence was truncated. Subsequently, non-linear fitting of the truncated data was performed to calculate the change in melting temperature (ΔTm) with a Boltzmann sigmoidal curve fitting (Sigmoidal dose-response) (Huynh and Partch, 2015). Changes in melting temperature were compared using a 1-way ANOVA.

## Results

### *In silico* Drug Discovery Identifies 12 Potential BFT Inhibitory Compounds

To identify molecules that bind to the active site of BFT, two complementary *in silico* drug discovery techniques were used; KRIPO and MOE. The KRIPO screen resulted in 476 small molecules that were predicted to bind to the (sub)pockets of BFT-3. Subsequently, the MOE-screening resulted in a selection of 43 compounds that interact with the pharmacophore description of the zinc atom. Finally, a total of 12 compounds, available in regular vendor catalogs, was purchased and screened *in vitro* (Table 1 and Supplementary Table 3).

Sequence alignment of the three BFT isoforms (Accession numbers NCBI: BFT-1: BAA77276, BFT-2: AAB50410, BFT-3: AAD33214) showed a high protein sequence identity (≥ 93%) among all isoforms (Supplementary Figure 3). For BFT-1 and BFT-2, a homology model was built with the automated protocol of YASARA (http://www.yasara.org/) using the x-ray structure of BFT-3 as template (Goulas et al., 2011). Overlaying BFT isoform structures reveal that residue 357 is the only residue in the active site that differs between every isoform. For residue positions 277, 280, 316, 319, 369, and 370, that also help frame the active site (Goulas et al., 2011), BFT-1 contains different amino acids, while BFT-2 and BFT-3 are identical (Supplementary Figure 3). The high similarity between the 3 BFT isoforms increases the likelihood that the 12 selected compounds interact with all isoforms, although they may not be equally effective due to small differences in the active site and biologic activity (Sears, 2001). Therefore, inhibition of biological activity was tested on all isoforms.

### Chenodeoxycholic Acid Inhibits Morphology Change by BFT-1 and BFT-3

**Figure 3:**
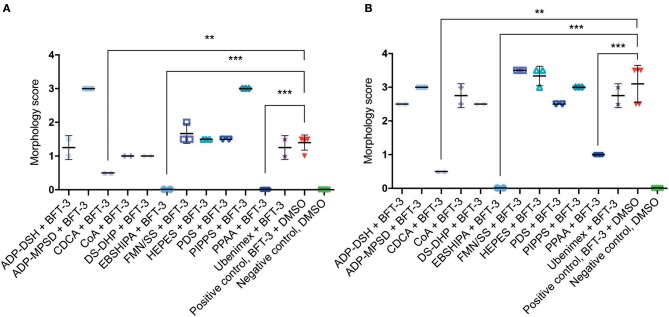
Morphological changes of *in silico* selected compounds. Drugs EBSHIPA, PPAA, and CDCA showed a 100%, 68–100%, and 64–84% reduction of BFT-3 related morphology changes, respectively, after 2 **(A)** and 3 **(B)** hours. *N* = 2–5 for each condition with (error) bars indicating means and standard deviations. (unpaired *t*-test, ^**^P < 0.01; ^***^P < 0.001).

To assess the efficacy of the 12 selected potential drug molecules, HT29/c1 cells were treated with BFT-3, in combination with each selected compound. Because the *in silico* drug design was based on the BFT-3 isoform, the initial screening focused on BFT-3 inhibition after 2- (Figure 3A) and 3-h (Figure 3B).

**Figure 4:**
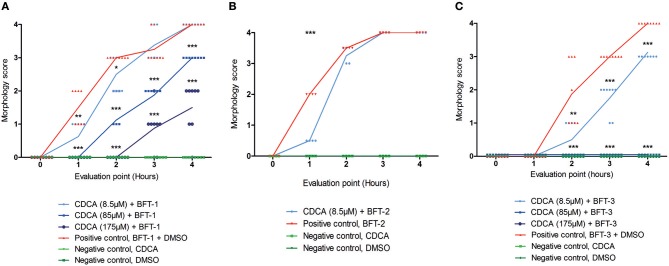
Morphological inhibition of BFT-1, BFT-2, and BFT-3 by CDCA. BFT-1, BFT-2, and BFT-3 related morphology changes were inhibited by CDCA, where an increase in CDCA concentration correlated with increased inhibition for BFT-1 and BFT-3. Depending on measurement times, CDCA at a concentration of 8.5, 85, and 175 μM resulted in 0–42%, 25–63%, and 73–100% inhibition of BFT-1 **(A)**. BFT-2 related morphology changes, only tested at a CDCA-concentration of 8.5 μM, were reduced by 75% **(B)**. BFT-3 was inhibited by CDCA at a concentration of 8.5, 85, and 175 μM with 25–73%, 100%, and 100% inhibition, respectively, depending on measurement times **(C)**. Negative control experiments did not display any morphology changes (score = 0 for all 8 replicates). Lines indicate means and *N* = 8 for each condition. Significance is marked by asterisks (unpaired *t*-test, ^*^P = 0.01–0.05; ^**^P = 0.001–0.01; ^***^P < 0.001).

Of all the selected compounds, only chenodeoxycholic acid (CDCA) resulted in significant reduction of HT29/c1 morphological change due to BFT-3. The inhibition peaked 3 h after addition of BFT-3 with a mean reduction in morphology score of 84% (minimum 80%, maximum 86%) compared to the positive control (*N* = 2, unpaired *t*-test, *P* = 0.0014). While EBSHIPA and PPAA also seemed to reduce BFT-3 related morphology changes, they were excluded from further testing due to their effects on cell morphology change. Both EBSHIPA and PPAA resulted in CEC cell cluster shrinkage and detachment of cells from the wells. These effects interfered with morphology scoring and hence discriminating between the effects of the compounds and BFT. Next, the BFT inhibitory effect of CDCA was tested at different concentrations (8.5, 85, and 175 μM) with BFT-1, BFT-2, and BFT-3. Increasing concentrations of CDCA inhibited morphology changes for both BFT-1 and BFT-3 (Figures 4A–C). While BFT-1 activity was only fully inhibited (identical to the negative control experiment) by CDCA at 175 μM for the first 2 h (*N* = 8, unpaired *t*-test, *P* < 0.0001), BFT-3 activity was fully inhibited by CDCA at 85 and 175 μM for 4 h compared to the positive control (*N* = 8, unpaired *t*-test, *P* < 0.0001). Less but still significant inhibition of morphological change was observed with lower concentrations of CDCA. CDCA at a concentration of 8.5 μM only showed inhibition in morphological assays with BFT-2 after 1 h. Due to the high potency of the BFT-2 concentrate and the associated difficulties in titration to similar levels as BFT-1 and BFT-3, we decided not to test CDCA at higher concentrations in assays with BFT-2 and to focus on BFT-1 and BFT-3.

### CDCA Reduces E-Cadherin Cleavage and IL-8 Secretion Induced by BFT-1 and BFT-3

**Figure 5:**
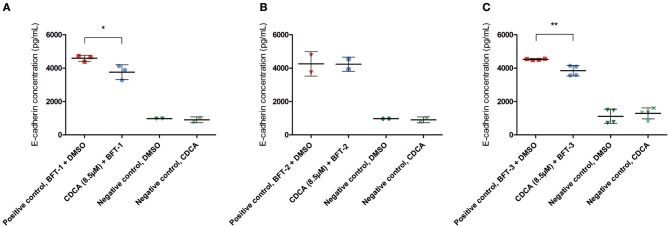
CDCA inhibits E-cadherin cleavage induced by BFT-1 and BFT-3. The release of E-cadherin into the cell medium supernatant for 3 h was measured with an E-cadherin ELISA. CDCA at 8.5 μM led to an average 18% (unpaired *t*-test, *P* = 0.0389) **(A)**, 0% **(B)**, and 15% (unpaired *t*-test, *P* = 0.005) **(C)** reduction of E-cadherin concentrations in morphology assays with BFT-1 **(A)**, BFT-2 **(B)**, and BFT-3 **(C)**, respectively. Bars indicate means and standard deviations. BFT-1, *N* = 2–3, BFT-2, *N* = 2, and BFT-3, *N* = 4 (unpaired *t*-test, ^*^P = 0.01–0.05; ^**^P = 0.001–0.01).

To better quantify the inhibitory action of CDCA on BFT biological activity, E-cadherin cleavage and IL-8 secretion were measured in cell supernatants. The amount of E-cadherin released in the supernatant is a measure for the activity of BFT, thus, effective inhibition of BFT by the selected inhibitors is predicted to reduce E-cadherin release. CDCA (8.5 μM) reduced E-cadherin release by 18% for BFT-1 (minimum 6%, maximum 30%, *N* = 2–3, unpaired *t*-test, *P* = 0.0389) and 15% for BFT-3 (minimum 8%, maximum 22%, *N* = 4, unpaired *t*-test, *P* = 0.005), when compared to the positive control (Figures 5A–C). This suggests that even at 8.5 μM CDCA, E-cadherin release by BFT-1 and BFT-3 is already inhibited. CDCA did not result in significantly reduced E-cadherin release by CECs exposed to BFT-2.

**Figure 6:**
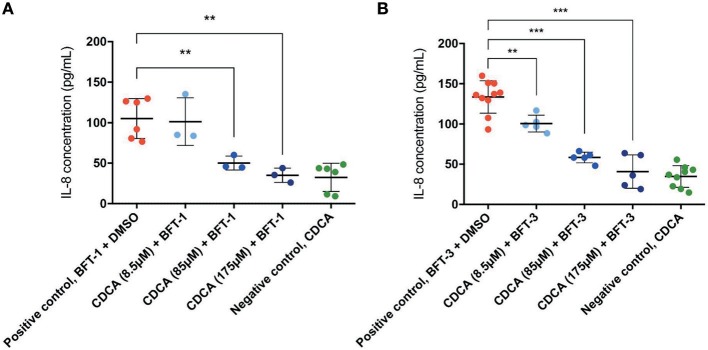
CDCA inhibits IL-8 secretion induced by BFT-1 and BFT-3. The amount of IL8 secreted by HT29/c1 cells for 4 h was measured by an IL-8 ELISA. CDCA in increasing concentrations shows significant inhibition of BFT-1 **(A)** and BFT-3 **(B)**. In experiments with BFT-1, CDCA results in a significant mean 52% (unpaired *t*-test, *P* = 0.0080) and 67% (unpaired *t*-test, *P* = 0.0023) reduction of IL-8 at 85 and 175 μM, respectively **(A)**. For BFT-3, CDCA significantly reduced IL-8 concentrations with a mean 24% (unpaired *t*-test, *P* = 0.0049), 56% (unpaired *t*-test, *P* < 0.0001) and 69% (unpaired *t*-test, *P* < 0.0001) at 8.5, 85, and 175 μM, respectively **(B)**. BFT-1, *N* = 3–6 and BFT-3, *N* = 5–10. Bars indicate means and standard deviations. (unpaired *t*-test, ^**^P = 0.001–0.01; ^***^P < 0.001).

Similar to E-cadherin release, effective inhibitors of BFT should lower the concentration of IL-8 in the cell culture supernatant after BFT-exposure. Indeed, CDCA significantly decreased IL-8 release of BFT-1 and BFT-3 treated HT29/c1 cells after 4 h. Furthermore, CDCA led to dose-dependent IL-8 reduction with CDCA at 175 μM yielding the highest rates, being 67% (minimum 43%, maximum 80%, *N* = 3–6, *P* = 0.0023) and 69% (minimum 32%, maximum 88%, *N* = 5–10, *P* < 0.0001) for BFT-1 and BFT-3 respectively (Figures 6A,B). At this peak reduction, IL-8 concentrations are almost equal to negative control values, indicating nearly full inhibition. The inhibition in E-cadherin release and IL-8 secretion correlates well with the observed morphological HT29/c1 cell changes induced by both BFT-1 and BFT-3 suggesting that inhibition of BFT by CDCA encompasses key aspects of BFT's effect on CECs.

### CDCA Interacts Directly With Recombinant BFT-1

**Figure 7:**
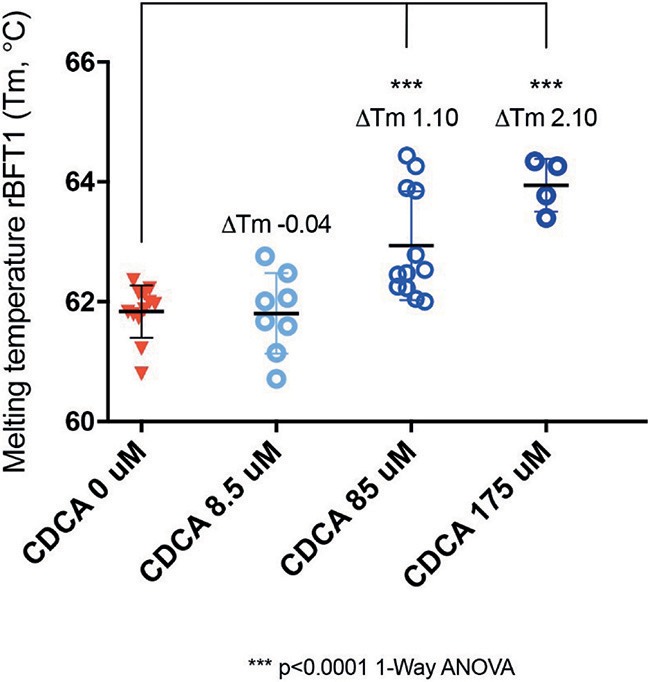
CDCA interacts directly with recombinant BFT-1. TSA with CDCA and rBFT-1 (*N* = 3), where CDCA resulted in a significant shift in melting temperature at concentrations of 85 and 175 μM (ΔTm 1.10 and ΔTm 2.10, respectively (*P* < 0.0001 for 1-Way ANOVA and Dunnett's multiple comparison test) when compared to the negative control condition (CDCA 0 μM). CDCA at a concentration of 8.5 μM did not result in a significant shift of the melting temperature of rBFT-1. Bars indicate means and standard deviations. *N* = 4–12 for each condition.

While it was encouraging that CDCA inhibited the activity of BFT-1 and BFT-3 in morphology assays and ELISA, these observations might still be the result of indirect effects (e.g., action on CECs) rather than direct binding of CDCA to BFT. To test whether the BFT inhibition is due to direct binding of CDCA to BFT, we performed a thermal shift assay (TSA) with purified rBFT-1. A TSA can be used to detect protein-ligand interactions. Proteins tend to have a more hydrophobic core and hydrophilic surface (Lodish et al., 2000). When heated, proteins unfold and their hydrophobic core is exposed. In a TSA, fluorescent dye is used to bind to the hydrophobic core and emit a measurable signal. corresponding to the melting temperature (Tm) of the protein. Protein-binding ligands increase the thermal stability and an increased Tm. A significant shift in melting temperature of rBFT-1 (62°C) was observed with concentrations of 85 and 175 μM CDCA (ΔTm 1.10, and ΔTm 2.10, *N* = 3, *P* < 0.0001, 1-Way ANOVA; Figure 7).

This indicates a direct interaction of CDCA with rBFT-1 that increases with the concentration of CDCA and corresponds to the inhibitory effect on the morphological assay for BFT-1 and CDCA. The raw fluorescent data used for calculation of ΔTm are presented in Supplementary Figure 4. These results strongly indicate that the two molecules form a physical bond, further supporting our *in silico* predictions. The predicted interaction of CDCA with the active site of BFT-3 is shown in Figure 1C.

## Discussion and Conclusions

The CRC-driving effect of ETBF is mediated by the toxin BFT, a metalloprotease that induces cleavage of E-cadherin resulting in marked changes in the biology of CECs. Here, we computationally predicted and screened a range of drug compounds for their ability to inhibit the proteolytic activity of BFT. Two compounds, EBSHIPA and PPAA, induced morphological effects on CECs resulting in cell cluster shrinkage and detachment. Because cell cluster shrinkage and detachment of cells interferes with morphology scoring and may be the reason of the observed BFT-inhibition they were excluded from further testing. We found that CDCA, a naturally-occurring primary bile acid, effectively inhibits BFT activity. Inhibition was observed in three complementary assays targeting different features of the cellular response to BFT-1 and BFT-3, including cell culture-based morphology change, E-cadherin cleavage, and IL-8 secretion. Additionally, direct interaction of CDCA to BFT was validated with a TSA.

The effect of CDCA on inhibition of the CEC response to BFT was different for the three BFT isoforms. CDCA most strongly inhibited BFT-3, than BFT-1, and only minimal inhibition was observed for BFT- 2. However, the inhibition of BFT-2 by CDCA was only tested at a concentration of 8.5 μM. This was due to the high potency of the BFT-2 concentrate and the associated difficulties in titration to similar levels as BFT-1 and BFT-3. Explanations for the difference in response to CDCA of the three BFT isoforms could be: (1) small differences in functional toxin concentration between isoforms, (2) the difference in amino acid sequence between isoforms may lead to altered protein folding or functionality of the binding pocket, and (3) the rate of activation of BFT by cleaving off the pro-domain and subsequent secretion may depend on the isoform or *B. fragilis* strain.

CDCA is a natural bile acid present in the human gut. Fast colonic transit is associated with higher concentrations of primary bile acids such as CDCA in the feces (Valdés Olmos et al., 1991). Delivery of CDCA via ileocolonic capsule accelerates colonic transit in healthy control subjects possibly by inducing pressure waves in the proximal colon (Bampton et al., 2002; Odunsi–Shiyanbade et al., 2010). Alternatively, loperamide, a drug used to reduce diarrhea, results in slower transit time and increases bile acid reabsorption, reducing primary bile acid concentrations in the feces. Natural CDCA concentrations range between 0.015 mM in healthy control subjects to 0.12 mM in patients with irritable bowel syndrome diarrhea. According to our results, the latter concentration is well within the range for BFT-inhibition by CDCA. Higher colonic transit due to diarrhea in these patients and hence higher fecal CDCA concentrations may thus target BFT activity (Peleman et al., 2017). Thus, natural CDCA concentrations could be a factor that modifies the outcome of ETBF-infection and colonization in individual patients, e.g., contributing to CRC or not.

An advantage of CDCA as a potential drug is that it is already being used as an oral treatment for gallstones, cerebrotendinous xanthomatosis, and constipation (Jiang et al., 2015; Fiorucci and Distrutti, 2019). This would simplify the transition from *in vitro* studies to *in vivo* and finally clinical studies, mainly because CDCA has already passed several toxicity tests (European Medicines Agency: Assessment report Chenodeoxycholic acid Leadiant) and has limited side effects at concentrations below 750 mg (Mok et al., 1974).

Aside from the well-known role of CDCA in lipid digestion in the bowel, CDCA is the strongest naturally occurring agonist of the farnesoid X receptor (FXR) (Makishima et al., 1999; Parks et al., 1999; Wang et al., 1999). FXR is expressed throughout the intestine and plays a pivotal role in the regulation of bile acid, lipid and glucose homeostasis, inflammatory responses, barrier function of the gut and prevention of bacterial translocation (Staels and Fonseca, 2009; Chai et al., 2015). Furthermore, FXR seems to play a thus far unexplained role in various diseases, including CRC, IBD and type 2 diabetes mellitus (Ding et al., 2015). This raises the question whether CDCA, while acting via FXR, could protect against CRC development, an idea that is partially supported by research suggesting that CDCA acts as an anti-inflammatory agent in the gut. Because CDCA directly binds to BFT (Wu et al., 2006), it may have a dual effect in altering ETBF colonic pathogenesis, both by binding to BFT directly as shown here, and by acting on FXR (Makishima et al., 1999; Parks et al., 1999; Wang et al., 1999).

This research was a first step to seek a compound that targets BFT to be used as putative preventive treatment for ETBF-related CRC and possibly other ETBF-related diseases. *In vivo* experiments are required to investigate the BFT-inhibitory effect of CDCA on tumorigenesis and inflammation in the colon and rectum. Eventually, clinical studies would be needed to determine which specific patient groups may benefit from treatment with CDCA.

In general, *in silico* drug screening serves as a very effective, time-saving and economical method applicable to research in which specific molecules are targeted. With an increasing performance of predicting drugs that interact *in vitro* and *in vivo, in silico* screening effectively complements laboratory screening methods.

## Data Availability Statement

The data that support the findings of this study are available from the corresponding author upon reasonable request.

## Author Contributions

PM performed experiments and wrote the manuscript. MT performed experiments. AB and BD designed the study, trained PM and MT, supervised the project, and wrote the manuscript. SW and CS trained and hosted PM and MT in their lab. AS created rBFT-1, MP, PM, and SH performed *in silico* modeling with KRIPO and MOE under the supervision of TR.

### Conflict of Interest

The authors declare that the research was conducted in the absence of any commercial or financial relationships that could be construed as a potential conflict of interest. The reviewer AG declared a past supervisory role with one of the authors CS to the handling editor.

## Footnotes

^1^Molecular Operating Environment (MOE), 2013.08; Chemical Computing Group ULC, 1010 Sherbooke St. West, Suite #910, Montreal, QC, Canada, H3A 2R7, 2018.
