# Supplementary material for: Drug Discovery and Repurposing Inhibits a Major Gut Pathogen-Derived Oncogenic Toxin
Source: Front Cell Infect Microbiol. 2019 Oct 25;9:364. doi: 10.3389/fcimb.2019.00364 (PMC6823872; doi:10.3389/fcimb.2019.00364)
Supplement: Supplementary file 1 [file Data_Sheet_1.PDF]

Supplementary Figure 1

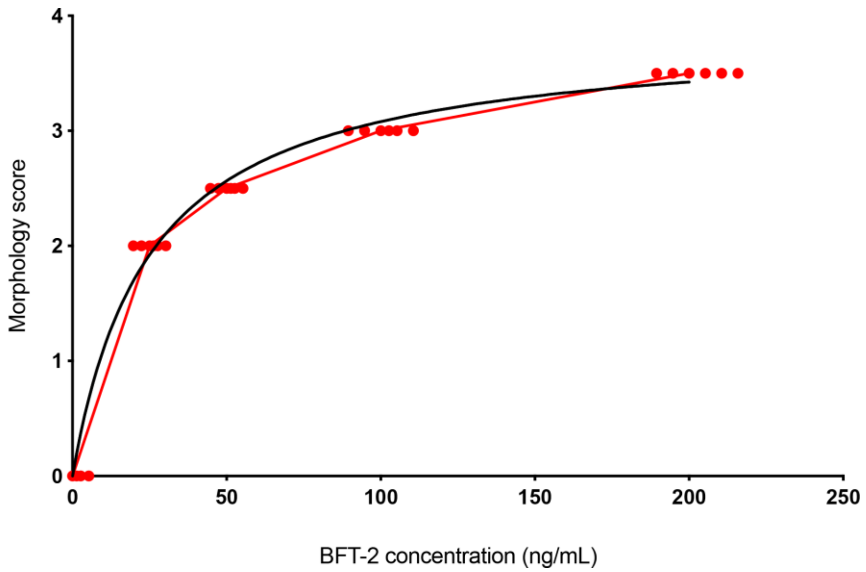

Standard curve of morphology assay scores in response to BFT-2 after 1 hour

This standard curve was fitted to morphology assay scores in response to known BFT-2 concentrations of pure BFT-2 protein using the Michaelis-Menten equation in GraphPad Prism version 6.00. For this study, BFT concentrations of crude toxin soups (BFT-1, BFT-2 and BFT-3) were calibrated by calculating interpolated concentrations using above standard curve from morphology assay scores of HT29/c1 cells exposed to BFT after 1 hour.

Supplementary Figure 2

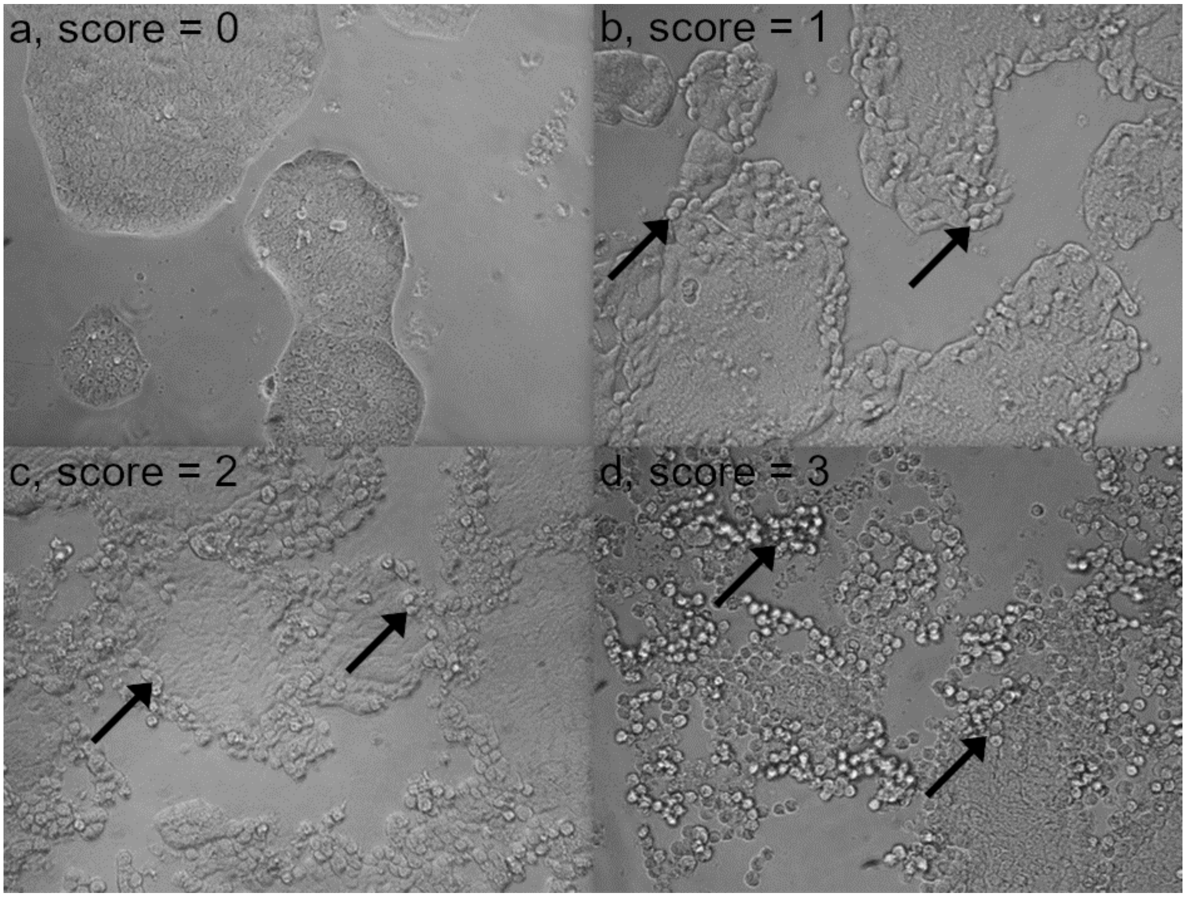

Morphological scoring system.

In the morphology assay scoring system, changes are scored on a scale from 0 to 4. 0: no change (a); 1: the cells at the edge of the cluster become rounded with visible sharp edges (b); 2: more than one layer of the cells at the outer surface of a cluster are changed (c); 3: some cells at the center of the clusters (usually larger clusters of cells) remain unchanged, the rest is affected (d); 4: all the cells are affected, one can barely see any normal cells (not shown).

Supplementary Methods 1

E-cadherin ELISA

A quantitative sandwich ELISA was performed to measure E-cadherin released into the supernatant (R&D Systems Human E-Cadherin DuoSet ELISA; Catalog no. DY648). Previous research has shown that E-cadherin is released at least during the first 3 hours of exposure to BFT29. Therefore, supernatants were taken from morphology assays after 3 hours of exposure, added to a 96-well plate coated with Human E-cadherin capture antibody within the linearity of the E-cadherin standard curve and processed according to the manufacturers instructions. E-cadherin concentrations were calculated using a 4-parameter logistic standard curve using GraphPad Prism version 6.00 as recommended by the manufacturer.

IL-8 ELISA

Supernatants were obtained from morphology assays 4 hours after initializing the experiment and added to a 96-well plate coated with IL-8 capture antibody within the linearity of the IL-8 standard curve (BD Pharmingen 554716) and then incubated for 2 hours at RT. After aspiration and washing with 0.05% Tween® 20 in PBS, wells were treated with IL-8 Biotinylated detection antibody (BD Pharmingen 554718), incubated for 1 hour and then washed. Wells were then incubated with streptavidin-HRP for 20 minutes and washed before treating with 1-Step™ Ultra TMB-ELISA Substrate Solution for 7 minutes. H2SO4 was used as to stop the reaction. Optical density was measured at 450 nm with a 655-nm reference wavelength in a microplate reader (Bio-Rad). IL-8 concentrations were calculated with a second-order polynomial (quadratic) standard curve using GraphPad Prism version 6.00 as recommended by the manufacturer30.

Recombinant BFT-1 production

Recombinant BFT-1 (rBFT-1) was generated with Promega simple step KRX competent cells (Catalog no. L3002) that were transformed with a Promega N-terminal Halo-tag vector (pH6HTN His6HaloTag® T7 vector) containing the bft-1 gene without promotor region. The N-terminal vector contains the HaloTag® fusion protein gene that links the HaloTag® fusion protein to the N-terminal side of BFT-1. Primers were designed to add restriction enzyme sites to bft-1 (Supplementary Table 2), enabling insertion into the Halotag vector. Additional primers were designed to confirm a correct insertion of the bft-1 gene. For eventual purification, the recombinant fusion protein was captured with HaloLink resin based on the manufacturer’s protocol. The ProTEV protease (Promega cat# V6101) was then used to release the BFT protein from the Halo Tag fusion protein. After removing the TEV protease using Ni-Resin, the protein eluate was used for the thermal shift assay.

Supplementary Figure 3

Aligned amino acid sequences of the 3 BFT isoforms.

1. = amino acid sequence of *bft-1*, VPI13784 Wt, accession number NCBI: BAA77276
2. = amino acid sequence of *bft-2*, 86–5443-2–2 Wt, accession number NCBI: AAB50410
3. = amino acid sequence of *bft-3*, K570 Wt, accession number NCBI: AAD33214

m = locations in amino acid sequence that contain a different amino acid in one or two of the other isoforms
m = segment of the pro domain we used for our comparison for the screening, see *material and methods*
m = these amino acids are part of BFT's active site, see *material and methods*
m = these amino acids constitute the C-terminal end of BFT, see *material and methods*

```
1. mknvklllml gtaallaacs neadslttsi dapvtasidl qsvsytdlat qlndvsdfgk      60
2. mknvklllml gtaallaacs neadslttsi dtpvtasidl qsvsytdlat qlndvsdfgk      60
3. mknvklllml gtaallaacs neadslttsi dapvtasidl qsvsytdlat qlndvsdfgk      60

1. miilkdngfn rqvhvsmdkr tkiqldnenv rlfngrdkds tsfilgdefa vlrfyrnges      120
2. miilkdngfn rqvhvsmdkr tkiqldnenv rlfngrdkds tsfilgdefa vlrfyrnges      120
3. miilkdngfn rqvhvsmdkr tkiqldnenv rlfngrdkds tnfilgdefa vlrfyrnges      120

1. isyiaykeaq mmneiaefya apfkktrain ekeafeciyd srtrsagkdi vsvkinidka      180
2. isyiaykeaq mmneiaefya apfkktrain ekeafeciyd srtrsagkdl vsvkinidka      180
3. isyiaykeaq mmneiaefya apfkktrain ekeafeciyd srtrsagkyp vsvkinvdka      180

1. kkilnlpecd yindyiktpq vphgitesqt ravpsepktv yviclrengs tiypnevsaq      240
2. kkilnlpecd yindyiktpq vphgitesqt ravpsepktv yviclresgs tvypnevsaq      240
3. kkilnlpecd yindyiktpq vphgitesqt ravpsepktv yviclrengs tvypnevsaq      240

1. mqdaansvya vhglkryvnf hfvlytteys cpsgdakegl egftaslksn pkaegyddqi      300
2. mqdaansvya vhglkrfvnl hfvlytteys cpsgnadegl dgftaslkan pkaegyddqi      300
3. mqdaansvya vhglkryvnl hfvlytteya cpsgnadegl dgftaslkan pkaegyddqi      300

1. yflirwgtwd nkilgmswfn synvntasdf easgmsttql mypgvmahel ghilgaehtd      360
2. yflirwgtwd nnilgiswld synvntasdf kasgmsttql mypgvmahel ghilgarhad      360
3. yflirwgtwd nnilgiswln synvntasdf kasgmsttql mypgvmahel ghilganhad      360

1. nskdlmyatf tqylshlsek nmdiiaknlg weaadgd                               397
2. dpkdlmysky tqylfhlsee nmyriaknlg weiadgd                               397
3. dpkdlmysky tqylfhlsek nmdiiaknlg weiadgd                               397
```

Supplementary Figure 4

a

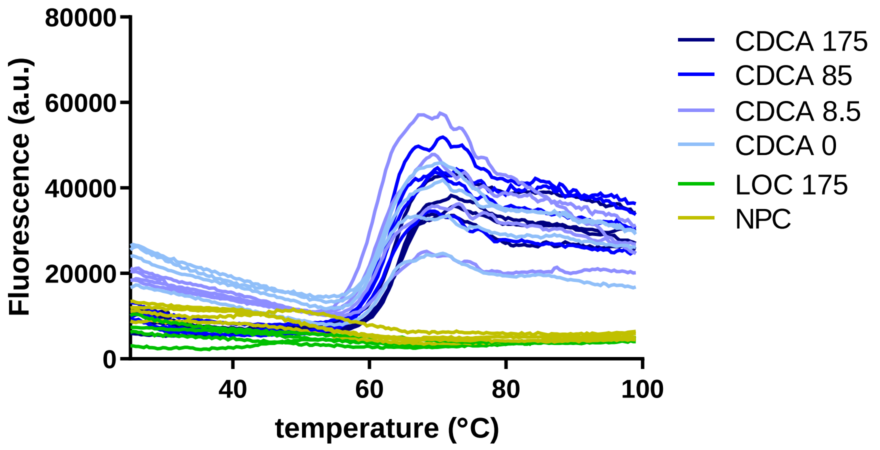

b

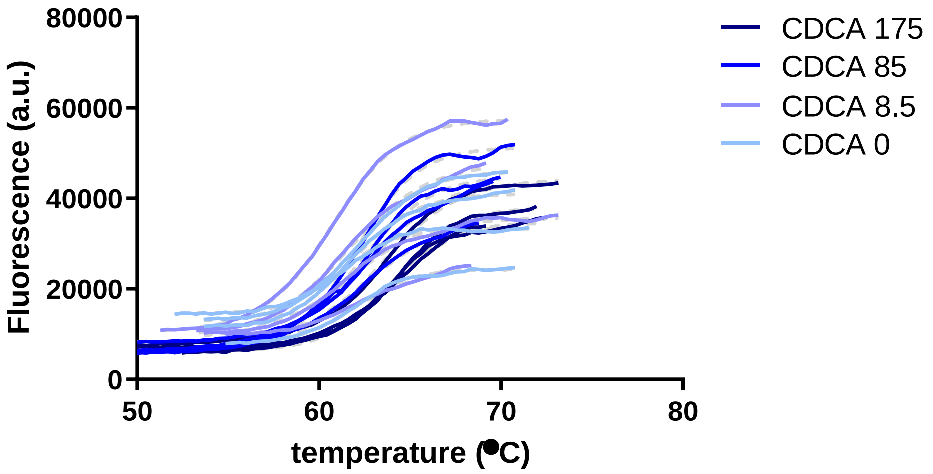

Thermal shift assay fluorescence levels.

In the thermal shift assay (TSA), fluorescence (in arbitrary units) was measured over an increasing temperature (a) for CDCA at 8.5, 85 and 175 µM. As reference a no protein control was used (NPC; containing only buffer, water and dye) and a ligand only control (LOC; containing CDCA at 175 µM, but no BFT). The post-peak truncated fluorescence data was deducted from the lowest and highest fluorescence level and used to calculate melting temperatures (b).

Supplementary Table 1

Composition of treatment solutions for experiments with BFT-3.

**Treatments**	**Compound (μM)**	**DMSO (%)**	**BFT-3 in dPBS** (pM)**	**dPBS** 1X (%)**
Compound + BFT-3*	8.5	0.085	100	0
Compound only	8.5	0.085	0	15
DMSO +BFT-3*	0	0.085	100	0
DMSO only	0	0.085	0	15
BFT-3*	0	0	100	0
Blank (DMEM + dPBS**1x)	0	0	0	15

*Calibrated concentrations of crude BFT were used in toxin assays with BFT-1, BFT-2 and BFT-3 at 200 pM, 300 pM and 100 pM concentrations, respectively.

**dPBS = Dulbecco's phosphate-buffered saline

Supplementary Table 2

Primers used to amplify bft gene with restriction enzyme sites attached at each end for insertion to vector

HaloN-bft-F_pvuI	GCGCGATCGAAATGAAGAATGTAAAG
HaloN-bft-R_pstI	CTGCTGCAGTTACTAATCGCCATCTGC

Primers used to amplify BFT gene for clone screening

bft 368F	GAACCTAAAACGGTATATGT
bft 368R	GTTGTAGACATCCCACTGGC

Primers used for DNA sequencing to confirm the bft gene insertion

Halo859-F	AAAAGCCTGCCTAACTGCAA
T7 terminator-R	TAGACTGGGCGGTTTTATGG

Supplementary Table 3

Twelve selected in silico putative fragilysin inhibiting compounds.

Identifier	Name, vendor and product number	Function	2D structure
ADP-DSH	Adenosine 5'-Diphosphate Disodium Salt Hydrate<br><br>Vendor: Tokyo Chemical Industry (TCI)<br><br>Product number: A0626	ADP, platelet activator	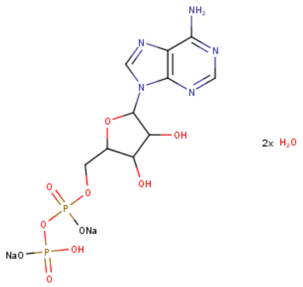
ADP-MPSD	Adenosine 5'-diphosphate monopotassium salt dihydrate<br><br>Vendor: Ambinter<br><br>Product number: Amb9809267	ADP, platelet activator	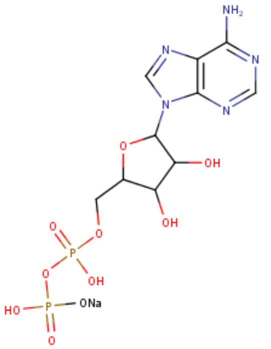
CDCA	Chenodeoxycholic acid<br><br>Vendor: Enamine<br><br>Product number: EN300-75328	Bile acid, primarily a medical therapy for dissolving gallstones	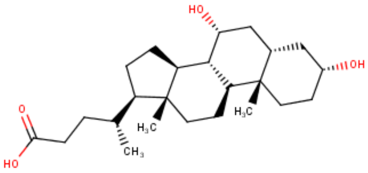
CoA	Coenzyme A hydrate, =85% (UV, HPLC)<br><br>Vendor: Sigma Aldrich	Metabolic cofactor in tricarboxylic acid cycle, and the synthesis and oxidation of fatty acids.	

	Product number: C4282		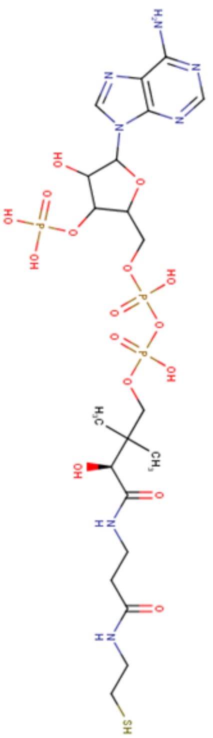
DS-DHP	Disodium 2,3-dihydroxypropyl phosphate<br><br>Vendor: Chembridge<br><br>Product number: 5106739	Phosphoric ester of glycerol, involved in reoxidation of NADH to NAD+ in mitochondria	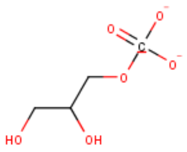
EBSHIPA	2-(4-Ethoxy-benzenesulfonylamino)-3-(1H-indol-3-yl)-propionic acid<br><br>Vendor: Enamine<br><br>Product number: EN300-00604	Possibly involved in platelet aggregation	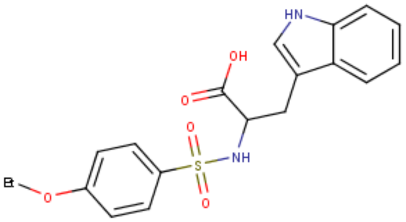
FMN/SS	Riboflavin 5'-Monophosphate Sodium Salt<br><br>Vendor: TCI	Prosthetic group (cofactor) used by oxidoreductase enzymes such as NADH dehydrogenase	

	Product number: R0023		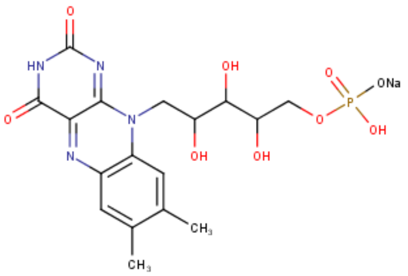
HEPES	2-[4-(2-Hydroxyethyl)-1-piperazinyl]ethane sulfonic Acid<br><br>Vendor: TCI<br><br>Product number: H0396	Zwitterion, used as buffering agent	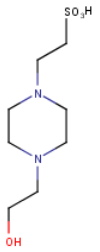
PDS	Phosphoramidon disodium salt<br><br>Vendor: Apollo Scientific<br><br>Product number: BIMI0549	Metallo-endopeptidase and metalloproteinase inhibitor, potent inhibitor of thermolysin	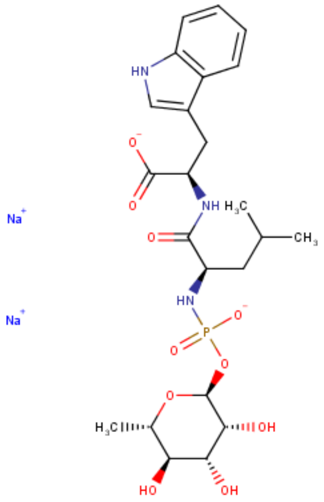
PIPPS	Piperazine-n,n'-bis(3-propanesulfonic acid)<br><br>Vendor: Ambinter<br><br>Product number: Amb2721614	-	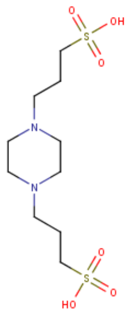

PPAA	[4-(1H-pyrazol-1-yl)phenyl]acetic acid<br><br>Vendor: Enamine<br><br>Product number: EN300-40902	-	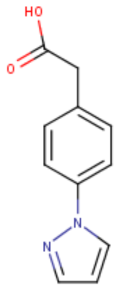
Ubenimex	Ubenimex<br><br>Vendor: Boerchem Pharmatech BB<br><br>Product number: BC219387	Competitive protease inhibitor, treatment of acute myelocytic leukaemia	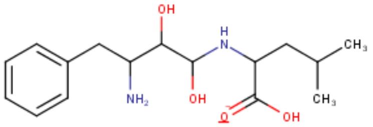
